# Characteristics and Trends of the Hospital Standardized Readmission Ratios for Pneumonia: A Retrospective Observational Study Using Japanese Administrative Claims Data from 2010 to 2018

**DOI:** 10.3390/ijerph18147624

**Published:** 2021-07-17

**Authors:** Ryo Onishi, Yosuke Hatakeyama, Kunichika Matsumoto, Kanako Seto, Koki Hirata, Tomonori Hasegawa

**Affiliations:** Department of Social Medicine, Toho University School of Medicine, 5-21-16 Omori-Nishi, Ota-ku, Tokyo 143-8540, Japan; ryo.onishi@med.toho-u.ac.jp (R.O.); yosuke.hatakeyama@med.toho-u.ac.jp (Y.H.); rakchart@med.toho-u.ac.jp (K.M.); setokana@med.toho-u.ac.jp (K.S.); koki.hirata@med.toho-u.ac.jp (K.H.)

**Keywords:** administrative claims data, Japan, pneumonia, patient readmission, quality indicator

## Abstract

Previous studies indicated that optimal care for pneumonia during hospitalization might reduce the risk of in-hospital mortality and subsequent readmission. This study was a retrospective observational study using Japanese administrative claims data from April 2010 to March 2019. We analyzed data from 167,120 inpatients with pneumonia ≥15 years old in the benchmarking project managed by All Japan Hospital Association. Hospital-level risk-adjusted ratios of 30-day readmission for pneumonia were calculated using multivariable logistic regression analyses. The Spearman’s correlation coefficient was used to assess the correlation in each consecutive period. In the analysis using complete 9-year data including 54,756 inpatients, the hospital standardized readmission ratios (HSRRs) showed wide variation among hospitals and improvement trend (r = −0.18, *p* = 0.03). In the analyses of trends in each consecutive period, the HSRRS were positively correlated between ‘2010–2012’ and ‘2013–2015’ (r = 0.255, *p* = 0.010), and ‘2013–2015’ and ‘2016–2018’ (r = 0.603, *p* < 0.001). This study denoted the HSRRs for pneumonia could be calculated using Japanese administrative claims data. The HSRRs significantly varied among hospitals with comparable case-mix, and could relatively evaluate the quality of preventing readmission including long-term trends. The HSRRs can be used as yet another measure to help improve quality of care over time if other indicators are examined in parallel.

## 1. Introduction

Pneumonia is one of the leading causes of morbidity, hospitalization, and mortality associated with infectious diseases worldwide and affects all age groups, especially elderly people. Many developed countries, such as Japan, are now dealing with a super-aged society, wherein multi-morbidity is a common scenario. In Japan, 133,121 people died from pneumonia, and the crude mortality rate was 107.2 per 100,000 population in 2018 [1].

In the past few decades, many efforts have been made to improve the quality of in-hospital care for pneumonia. Indicators of the quality of pneumonia care include in-hospital mortality, readmission, and length of stay (LOS) [2,3,4]. In-hospital mortality from common diseases showed considerable variations, suggesting the potential for outcome improvement [2]. Previous studies indicated that optimal care for pneumonia during hospitalization might reduce the risk of in-hospital mortality and subsequent readmission which was considered an indicator of inadequate hospital care [5,6]. The hospital standardized mortality ratio (HSMR) is an indicator for assessing the in-hospital mortality ratio based on the case-mix adjustment with patient risk factors, and identifying areas that can be changed to improve patient safety and the quality of care. The HSMR has proven useful in many developed countries and areas, such as the USA, UK, Canada, Sweden, Australia, France, Singapore, and Hong Kong [7,8]. Recently, interest in preventing readmission has been growing worldwide.

In the USA, the hospital readmission reduction program (HRRP) started in 2013. This program aimed to establish a method for calculating the expected readmission rate and to create a financial penalty system for hospitals with excessive readmissions. Age, sex, and comorbidities were used for risk adjustment in the HRRP. Subsequent studies have reported positive effects such as a reduction in readmission rates and medical costs, with the introduction of the HRRP [9,10,11,12,13]. Moreover, it was revealed that the difference in hospital type or hospital location influenced the readmission rate [14,15,16,17,18,19,20]. In the HRRP, readmission was defined as patients who had been discharged and admitted again to the same or another hospital within 30 days by all-cause [21,22].

In Japan, in the diagnostic procedures combination (DPC) and per-diem payment system (PDPS), readmission to the same hospital because of the same disease within 7 days is regarded as a single continuous admission. The DPC/PDPS is a reimbursement system for acute care hospitals introduced in 2003, and the DPC database is a national administrative claim and discharge abstract database for inpatient acute care [23,24]. Administrative data, such as DPC data, have been used in designing health policies, disease management, and analysis of healthcare processes and patient outcomes [25,26]. Each hospital can analyze its own quality of care using DPC data. Additionally, the Japanese public medical insurance system began to reimburse a discharge support plan and a specialist team conference for preventing readmission in 2016.

It is important for hospitals to recognize the specific conditions and procedures that significantly affect the lives of patients in the Japanese health care system. Hospitals need to improve patient outcomes and construct the plan-do-check-act (PDCA) cycle for improving the quality of care. In the process for improving quality of preventing readmission, each hospital needs to objectively grasp the quality of care, and it is necessary to construct the standardized quality indicators with patient risk adjustments, and to consider the quality of care compared to other hospitals. The hospital standardized readmission ratio (HSRR) indicator can measure risk-adjusted readmission ratio by considering factors known to affect the risk of readmission. The HSRR indicator provides incentives for investing in interventions to improve in-hospital care and the readiness of patients for discharge for preventing readmission.

This study aimed to reveal the trends of the hospital standardized readmission ratios (HSRRs) for pneumonia in Japan. To the best of our knowledge, this is the first large-scale study calculating the HSRRs for pneumonia in Japan and revealing a 9-year trend using administrative data.

This study was constructed the following sections. First, the data introduction, analytical models, comparisons with other indicators, and statistical analysis methods were introduced in the methods section. Second, the results section shows the analysis results of two HSRR models and comparison of indicators between mortality and readmission. Third, based on the results, the discussion section presented the features of the models, new discoveries in this study, limitations, and future research. Finally, we presented the conclusion based on the entire study outcomes.

## 2. Methods

This was a retrospective observational study using the DPC database. We analyzed the HSRR using the variables and risk-adjusted methodology following the previous studies [3,21,27,28,29]. In previous studies, risk-adjusted readmission ratio studies using big data were conducted mainly in the USA. Among those studies, risk-adjustments were made according to age, sex, and comorbidities, but we considered that the risk-adjustments for severity and condition at discharge were insufficient. As an analysis of quality indicators using DPC data in Japan, there was a study of standardized mortality ratio. Based on this study, we examined the variables needed to calculate the standardized readmission ratio. Additionally, we included other variables related to long-term hospitalization and environment of after discharge.

### 2.1. Data Sources

DPC data of the Medi-Target benchmarking project managed by the All Japan Hospital Association (AJHA) were used. The AJHA is one of the largest nation-wide hospital associations in Japan, comprising 2500 hospitals, which manages the Medi-Target project, a benchmark project using clinical indicators based on DPC data. Participation in the Medi-Target project was optional, and there were 182 participating acute care hospitals which were small or medium-sized in 2010, submitting about 500,000 claims data annually [23]. In the Medi-Target benchmarking project, participating hospitals submit the DPC data to the AJHA, and the AJHA analyze it for activities to improve the quality of hospital care using quality indicator.

All hospital admissions with principal diagnosis of pneumonia from 2010 to 2018 were identified from the DPC database. The 10th revision of the International Statistical Classification of Diseases and Related Health Problems (ICD-10) codes (J12–18, J69, B01.2, B05.2, B59) were used to determine the diagnosis [26]. Hospitals with no discharged pneumonia patients in a year in the 9-year analysis or no readmission for pneumonia in each 3-year period were excluded.

Patients aged 15 years and over was included in this study. The patient data included age, sex, urgency of admission, Charlson comorbidity index (CCI), A-DROP score, length of stay (LOS), discharge destination, and readmission. Age, sex, urgency of admission, and CCI were used in a previous HSMR study [26]. Age was categorized according to the DPC/PDPS category: 15–64, 65–74, 75 and over. We adopted LOS and discharge destination as proxy variables for the patient conditions during hospitalization and after discharge from the DPC database.

The A-DROP scoring system, developed by the Japanese Respiratory Society (JRS), is a modified version of the CURB-65 scoring system. This system has a higher level of discrimination than CURB-65 or the pneumonia severity index, with a reported c-statistic of 0.85 [30,31]. It is a 6-point scale (0–5) that assesses the following parameters: (i) age (male ≥ 70 years, female ≥ 75 years); (ii) dehydration (BUN ≥ 210 mg/L); (iii) respiratory failure (SaO2 ≤ 90% or PaO2 ≤ 60 mmHg); (iv) orientation disturbance (confusion); and (v) low blood pressure (systolic blood pressure ≤ 90 mmHg). The severity of pneumonia is stratified into the following four levels according to the A-DROP score: (i) 0 as 0: mild; (ii) 1–2 as 1: moderate; (iii) 3 as 2: severe, and (iv) 4–5 as 3: extremely severe [32]. The CCI (range 0 to 3) derived from the secondary ICD-10 diagnosis codes was calculated. The CCI is a weighted score based on the number and type of diagnoses reported in the hospital summary data [33]. The CCI was calculated based on Quan’s modification [34]. LOS was adopted as a variable to adjust for the deterioration of physical function due to long-term hospitalization.

This study was based on a secondary analysis of administrative claims data. Owing to the anonymous nature of the data, no institutional review board (IRB) approval was needed for this kind of study in Japan [35]. This study was judged as not applicable for the ethical review by the Ethics Committee of Toho University School of Medicine (No. A19053).

### 2.2. Calculation of HSRRs

The HSRR was defined as the ratio of the actual number of readmissions to the expected number of readmissions multiplied by 100. Readmission was defined as a repeat admission due to pneumonia to the same hospital within 30 days from discharge. We set the definition of 30 days with reference to the HRRP in the USA, assuming long-term readmission that has not yet been introduced incentives for preventing readmission in Japan. In addition, risk adjustments were made to enable objective comparison of quality for preventing readmission care between hospitals of different sizes and functions. Moreover, the ratio is multiplied by 100 to make it easy for the hospital to appreciate its quality [7,8].

$$\mathrm{HSRR}=\left(\frac{\mathrm{Observed}\;\mathrm{number}\;\mathrm{of}\;\mathrm{readmissions}}{\mathrm{Expected}\;\mathrm{number}\;\mathrm{of}\;\mathrm{readmissions}}\right)\times 100$$

The observed number of readmissions is the sum of the actual number of readmissions to the same hospital, and the expected number of readmissions is the sum of the probabilities of readmissions. An HSRR above 100 indicates that the readmission ratio is higher than the overall average.

Multivariable logistic regression analysis was constructed to predict the chance of readmission for each patient with patient-level factors. Logistic regression analyses were performed to calculate the intercept of the covariates. The covariates for case-mix adjustment were age, sex, urgency of admission, A-DROP score, CCI, LOS, and discharge destination. Coefficients derived from the logistic regression analysis were used to calculate the probability of readmission. The sum of the predicted probabilities of readmissions (range, 0 to 1) provided the total expected number of readmissions in that hospital. The ratio of the expected number of readmissions and the actual number of readmissions provided the standardized ratio for that hospital of interest.

We considered two HSRR analyses. The first included hospitals that had at least 1 readmitted pneumonia patient in each period (2010–2012, 2013–2015, and 2016–2018) because hospitals with no readmission could not be applied to the HSRR, which was calculation as a ratio. The number of hospitals was different in each period. The analysis using the 3-year data was used for assessing the hospital trends in each consecutive period using each period data. The second analysis was the 9-year analysis, which was created to assess the trend of the HSRRs over time by including hospitals with complete data for nine years from 2010 to 2018. Fitting data from all nine years into one analysis allowed us to make valid comparisons over time.

### 2.3. Relationship between Readmission and In-Hospital Mortality

To assess the relationship between readmission and in-hospital mortality, we analyzed the correlation between the HSRR and HSMR of each hospital using the 9-year analysis. We calculated the HSMR using the same DPC database. The HSMR was defined as the ratio of the sum of actual number of in-hospital deaths to the expected number of in-hospital deaths multiplied by 100. In a study of HSMR, patient data were collected as variables to adjust for risks of in-hospital death using a multivariable regression analysis. Logistic regression analysis was performed to calculate the intercept of the covariates. Covariates were determined for the case-mix adjustment of patient-level data. The patient-level data included sex; age; urgency of admission (emergency or planned); mode of transportation (ambulance use); comorbidities on admission (CCI); age, dehydration, respiratory failure, orientation disturbance, and blood pressure (A-DROP) score on admission; operative status (surgery completed or not); and in-hospital death. The difference in variables between the HSRR and the HSMR was that the HSRR included LOS, discharge destination, excluded mode of transportation, and operative status. Coefficients derived from the logistic regression analysis were used to calculate the probability of in-hospital deaths.

$$\mathrm{HSMR}=\left(\frac{\mathrm{Observed}\;\mathrm{number}\;\mathrm{of}\;\mathrm{deaths}}{\mathrm{Expected}\;\mathrm{number}\;\mathrm{of}\;\mathrm{deaths}}\right)\times 100$$

### 2.4. Statistical Analysis

The following variables were examined for their association with readmission (HSRR): age, sex, urgency of admission, A-DROP score, CCI, LOS, and discharge destination. Regarding coefficient values, age, sex, urgency of admission, A-DROP score, CCI, and discharge destination were categorical variables; and LOS was expressed in days. The patient characteristics were compared using chi-square tests for categorical variables and t-tests for continuous variables. Continuous variables were summarized using descriptive statistics (mean ± standard deviation (SD)), whereas categorical variables were summarized as frequencies and proportions. In the analysis using the 9-year data, the 95% confidence interval (CI) of the HSRRs was calculated using Byar’s approximation.

The HSRRs were classified into two groups (HSRR ≤ 100 and HSRR > 100), and we investigated the change in the proportion of hospitals with good results (HSRR ≤ 100). The correlation between the HSRRs for each period in the analysis using the 3-year data was evaluated using Spearman’s correlation coefficient. The trend of the HSRRs from 2010 to 2018 was calculated using the mean HSRR and the proportion of the HSRR ≤ 100 hospitals in each period. The correlation between the HSRR and the HSMR was evaluated using Spearman’s correlation coefficient.

All statistical analyses were performed using the Statistical Package for the Social Sciences (IBM, Armonk, NY, USA, version 27.0.0). *p* values < 0.05 were considered to indicate statistical significance.

### 2.5. Patient and Public Involvement

This research was designed without patient involvement.

## 3. Results

### 3.1. Characteristics of the Study Population

After exclusions, the sample size of the 9-year data was 54,756 discharged patients with a primary diagnosis of pneumonia. In the analysis using the 9-year data, 85.0% of discharged patients were elderly (age ≥ 65 years). The analysis using the 3-year data comprised 51,107, 36,389, and 30,506 discharges in 167,120, and 83 hospitals in 2010–2012, 2013–2015, and 2016–2018, respectively. Table 1 shows the demographic characteristics of the overall sample.

In the analysis using the 9-year data, 3.4% of discharged patients were readmitted. The mean age of the readmitted patients was 79.5 years, 63.5% of these patients were male, the mean LOS was 24.8 days, and 62.7% of the patients were discharged home. Chronic pulmonary disease was the most common comorbidity, observed in 20.2% of the patients at admission. The other associated comorbidities at admission included congestive heart failure (16.6%), diabetes mellitus without chronic complication (13.3%), cerebrovascular disease (12.8%), and dementia (11.3%).

### 3.2. Characteristic of the HSRRs

Table 2 shows the coefficients and significance of the variables in the 9-year data. All included variables showed a significant relationship with readmission. In the analysis of the 9-year data, readmission was positive related to age, sex (male), CCI, A-DROP score, and LOS. These variables increased the risk of readmission for patients. Meanwhile, urgency of admission (emergency) and discharge destination (home) were negatively related with readmission. The 3-year data analyses were performed using the data for each period to verify the validity of the variables and models used (Table S1). The 9-year data analysis showed an internal consistency of the results.

**Figure 1:**
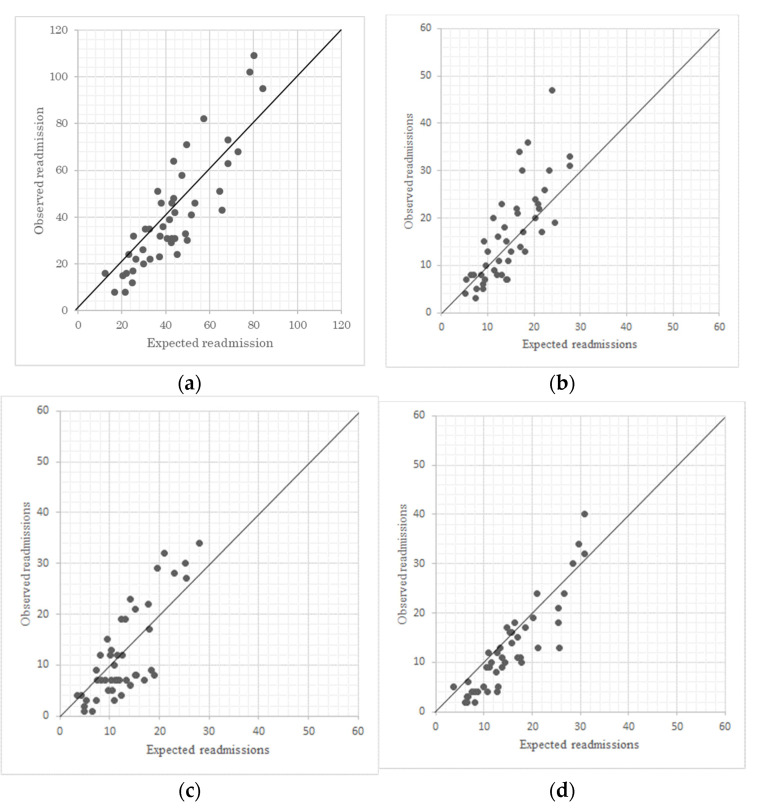
Variation of the HSRRs; (**a**). 9-year model; (**b**) 3-year model (2010–2012); (**c**). 3-year model (2013–2015); (**d**). 3-year model (2016–2018).

The HSRR varied widely across the hospitals included in this study. Figure 1 shows the variation in mean-SD of the HSRR in each period. In the analysis using the 9-year data, the HSRR ranged from 37.38 to 147.03 (92.15 ± 28.59). In the analysis using the 3-year data, the HSRRs ranged from 23.20 to 630.63 (106.98 ± 71.66), 16.26 to 508.47 (99.56 ± 65.18), and 12.11 to 623.15 (136.27 ± 86.20) in 2010–2012, 2013–2015, and 2016–2018, respectively. 

The analysis using the 9-year data, the HSRR was not correlated with the HSMR (r = 0.026, *p* = 0.894).

### 3.3. Trends of the HSRRs

The analysis using the 9-year data, a continuous trend of the HSRRs was found. The mean HSRR decreased by 107.99, 87.38, and 76.67 in 2010–2012, 2013–2015, and 2016–2018, respectively. The number of HSRR ≤ 100 hospitals increased: 21 (46.7%), 27 (60.0%), and 34 (75.6%) in 2010–2012, 2013–2015, and 2016–2018, respectively.

In the analysis using the 3-year data, the HSRRs in the first (2010–2012) and second (2013–2015) periods had a significantly positive correlation (r = 0.255, *p* = 0.010), and the HSRRs in the second (2013–2015) and third (2016–2018) periods had a significantly positive correlation (r = 0.603, *p* < 0.001). This indicated that high/low HSRR hospitals were likely to continue over time (Table 3).

## 4. Discussion

This study denoted that the HSRRs could be calculated using DPC data in Japan. The methodology has proposed is highly novel in that it examines the risk-adjusted readmission ratio using big data, which has not yet been established in Japan. Furthermore, we believe that utilizing the DPC/PDPS data introduced by most acute care hospitals in Japan is highly universal and can contribute to further research. In practice, all acute care hospitals are requested to submit the DPC data to the government, and the HSRR can be used to assess the quality of inpatient care for pneumonia. The results showed that the HSRRs of pneumonia varied considerably among hospitals with comparable case-mixes. In the analysis using the 9-year data, the hospital with the highest HSRR had a value 3.93 times higher than that of the hospital with the lowest HSRR. After adjustment for case-mixes, some hospitals were found to have a higher readmission ratio.

In the analysis using the 9-year data, all variables were related to readmission. However, readmission might associate with other confounders, which were not include in DPC database, such as the treatment environment after discharge. In particular, emergency admission and discharge to home were negatively related to readmission. Pneumonia patients with a planned admission were more likely to have a chronic disease and were more likely to be readmitted than those with an emergency admission, who required sudden and acute care. Similarly, it can be inferred that patients who are able to return home after discharge have a low risk of secondary pneumonia based on their condition and the care system at home. As for patient age, 85.0% of the discharged patients were elderly, and older age contributed to the increase in the readmission ratio. It is well known that ageing contributes to the aggravation of pneumonia, and, thus, our results are consistent with those of previous studies [20,29,31]. As for sex, male patients were more likely to be readmitted. Collectively, pneumonia is more likely to become severe at a younger age in men than in women [31]. As for LOS, we found that the longer the LOS, the higher the possibility of readmission. For elderly patients, long-term hospitalization has a high risk of deteriorating the activities of daily living (ADL) score and a high risk of dementia. Further, immunity and swallowing function also decrease, which increases the risk of readmission with secondary pneumonia. LOS is also used as an indicator of the quality of in-hospital care [3]. With respect to the severity of pneumonia, the higher the A-DROP score, the greater the probability of readmission. This indicated that patients with more severe pneumonia need more attention at the time of discharge.

As for the trend of the HSRRs, an improving tendency was confirmed in this study. The number of hospitals with an HSRR ≤ 100 increased, and the mean HSRRs decreased for three consecutive periods. This result may indicate the hospitals’ efforts in preventing readmission. The readmission prevention system has just been implemented in Japan, and a long-term study on this aspect is needed in the future. In this study, there was no correlation between the indicator for the risk-adjusted readmission ratio and in-hospital mortality ratio in each hospital. This may suggest that the HSRR and HSMR reflect differences in in-hospital care. This finding indicated that HSMRs represented the quality of in-hospital care in preventing death, whereas the HSRRs may reflect the composite quality of overall in-hospital care, discharge support, and post-discharge care.

The most remarkable finding of this study was the correlation analysis outcome: hospitals with low HSRR were likely to produce the same results in the following period and vice versa. This result indicated that the HSRR is a stable index, and hospitals with a high HSRR could benefit from appropriate support. It is important for hospitals to be aware of the quality of their medical care, and appropriate measures should be implemented. Therefore, we considered the HSRR as a quality indicator of in-hospital care based on the DPC data, which is standardized nation-wide. DPC data mainly include information on admission, and there is limited information regarding patient status at the time of discharge limiting the predictive value of the analysis. In the future, it will be important to modify the DPC/PDPS to include patient information at the time of discharge.

The strengths of this study include its large sample size, and a relatively long study duration of nine years. This study had some limitations. First, because this was a secondary analysis of administrative data, other factors might have influenced the expected readmission ratio [36]. Thus, adherence to the JRS guidelines, such as ‘commencement of antibiotic therapy within 4 h of admission’ could not examined, for example. Second, the sample population was derived from hospitals that voluntarily participated in this benchmarking project, and the survey population may not be representative of the entire population in Japan. Another limitation is the lack of data on readmission to another hospital, rehabilitation, care in home, and 30-day mortality after discharge. Moreover, it was unclear whether these readmissions were transfers from other hospitals. Each of these limitations might act as a potential confounder.

## 5. Conclusions

The results of this study suggested that it is possible to use data from the DPC database to compute the HSRRs. Importantly, the HSRRs of pneumonia varied significantly among hospitals with comparable case-mixes. During the study period, there were improving trends of the HSRR and the correlation between the HSRRs in consecutive period was observed. The HSRR, a new risk-adjusted quality indicator constructed in this study, might contribute to the study of the quality of hospital care. Preventing readmission is necessity for reducing social burden. The HSRR might give a basis for hospital administrators and policy makers to consider how to a way of preventing readmission. Future studies will be to elaborate the HSRR with data not available in this study.

## Figures and Tables

**Table 1:** Patient demographic characteristics in the analysis using the 9-year data *.

Characteristics		2010–2018 *(n* = 54,756)		
		Readmission	Non-Readmission	*p*
Demographic features				
Age 15–64 year (reference)		168 (0.3)	8137 (14.9)	<0.001 ^††^
Age 65–74 year		301 (0.5)	8394 (15.3)	
Age 75 year+		1377 (2.5)	36,379 (66.4)	
Sex (% of male)		1173 (2.1)	28,944 (50.1)	<0.001 ^††^
Comorbidity				
CCI score 0 (reference)		758 (1.4)	25,307 (46.2)	<0.001 ^††^
CCI score 1–2		799 (1.5)	21,376 (39.0)	
CCI score 3–4		250 (0.5)	5415 (9.9)	
CCI score 5+		39 (0.1)	812 (1.5)	
Urgency of admission (% of Emergency admission)		1546 (2.8)	46,218 (84.4)	<0.001 ^††^
Severity status				
ADROP score 0 (reference)		155 (0.3)	7956 (14.5)	<0.001 ^††^
ADROP score 1–2 (moderate)		1255 (2.3)	34,100 (62.3)	
ADROP score 3 (severe)		322 (0.6)	8186 (14.9)	
ADROP score 4–5 (extremely severe)		114 (0.2)	2668 (4.9)	
LOS (days)	mean ± SD	24.8 ± 24.7	20.2 ± 24.2	<0.001 ^†^
Discharge destination (home)		1157 (2.1)	36,192 (66.1)	<0.001 ^††^

* () values are % of 54,756; ^†^ *t*-test; ^††^ Chi-square test; CCI = Charlson comorbidity index, LOS = length of stay; *p* = two-tailed significance.

**Table 2:** Variables for the logistic regression analysis using the 9-year data.

	Coefficient	*p*	Odds Ratio	(95% CI)
Age 15–64 year (reference)				
Age 65–74 year	0.318	0.003	1.375	(1.117–1.692)
Age 75 year+	0.303	0.004	1.354	(1.103–1.663)
Sex (male)	0.357	<0.001	1.429	(1.295–1.577)
CCI score 0 (reference)				
CCI score 1–2	0.313	0.003	1.169	(1.055–1.294)
CCI score 3–4	0.315	<0.001	1.371	(1.182–1.589)
CCI score 5+	0.367	0.029	1.444	(1.038–2.010)
Urgency of admission (Emergency admission)	−0.324	<0.001	0.723	(0.637–0.821)
ADROP score 0 (reference)				
ADROP score 1–2 (moderate)	0.364	0.001	1.439	(1.163–1.781)
ADROP score 3 (severe)	0.361	0.003	1.435	(1.126–1.829)
ADROP score 4–5 (extremely severe)	0.424	0.004	1.528	(1.146–2.035)
LOS (days)	0.003	<0.001	1.003	(1.002–1.004)
Discharge destination (home)	−0.214	<0.001	0.807	(0.730–0.893)

CCI = Charlson comorbidity index, LOS = length of stay; *p* = two-tailed significance.

**Table 3:** Correlation between the HSRRs in each consecutive period (3-year analysis).

Period	*n*	r	*p*
2010–2012—2013–2015	99	0.255	0.010
2013–2015—2016–2018	80	0.603	<0.001

*n* = number of hospitals; r = correlation coefficient (Spearman’s non-parametric correlation); *p* = two-tailed significance.; Positive correlation coefficient means that hospitals with lower/higher HSRRs are likely to produce the same results in the following year.

## Data Availability

The data that support the findings of this study are available from (All Japan Hospital Association) but restrictions apply to the availability of these data, which were used under license for the current study, and so are not publicly available. Data are however available from the authors upon reasonable request and with permission of (All Japan Hospital Association).

## Supplementary Materials

The following are available online at https://www.mdpi.com/article/10.3390/ijerph18147624/s1, Table S1: Variables for the logistic regression analysis using the 3-year data.
