# Supplementary material for: Characteristics and Trends of the Hospital Standardized Readmission Ratios for Pneumonia: A Retrospective Observational Study Using Japanese Administrative Claims Data from 2010 to 2018
Source: Int J Environ Res Public Health. 2021 Jul 17;18(14):7624. doi: 10.3390/ijerph18147624 (PMC8304042; doi:10.3390/ijerph18147624)
Supplement: Supplementary file 1 [file ijerph-18-07624-s001.zip › ijerph-1303076-supplementary.pdf]

Table S1: Variables for the logistic regression analysis using the 3-year data

	Period 1 (2010-2012)		Period 2 (2013-2015)		Period 3 (2016-2018)	
Variables	Coefficient	Odds ratio (95% CI)	Coefficient	Odds ratio (95% CI)	Coefficient	Odds ratio (95% CI)
Age 15-64 year (reference)						
Age 65-74 year	0.522	1.686 (1.374-2.068)	0.375	1.455 (1.129-1.874)	0.494	1.638 (1.200-2.237)
Age 75 year+	0.546	1.727 (1.412-2.112)	0.340	1.405 (1.089-1.814)	0.423	1.526 (1.122-2.076)
Sex (male)	0.275	1.316 (1.200-1.443)	0.340	1.405 (1.239-1.592)	0.520	1.682 (1.458-1.940)
CCI score 0 (reference)						
CCI score 1-2	0.091	1.095 (0.995-1.205)	0.317	1.374 (1.204-1.567)	0.348	1.416 (1.216-1.649)
CCI score 3-4	0.210	1.234 (1.072-1.420)	0.514	1.673 (1.395-2.006)	0.536	1.709 (1.398-2.089)
CCI score 5+	-0.041	0.960 (0.673-1.370)	0.228	1.256 (0.786-2.008)	0.705	2.024 (1.375-2.980)
Urgency of admission (Emergency admission)	-0.419	0.658 (0.601-0.720)	0.288	1.333 (0.910-1.953)	0.173	1.189 (0.780-1.813)
ADROP score 0 (reference)						
ADROP score 1-2 (moderate)	0.301	1.351 (1.097-1.664)	0.187	1.205 (0.936-1.553)	0.336	1.399 (1.017-1.925)
ADROP score 3 (severe)	0.249	1.283 (1.015-1.621)	0.136	1.145 (0.852-1.539)	0.263	1.301 (0.911-1.860)
ADROP score 4-5 (extremely severe)	0.589	1.803 (1.401-2.320)	0.104	1.110 (0.764-1.612)	0.061	1.063 (0.678-1.667)
LOS (days)	0.004	1.004 (1.003-1.005)	0.005	1.005 (1.003-1.006)	0.002	1.002 (1.000-1.004)
Discharge destination (home)	-0.284	0.753 (0.684-0.828)	-0.180	0.836 (0.732-0.954)	-0.287	0.751 (0.651-0.866)

CCI= Charlson comorbidity index, LOS= length of stay

p = two-tailed significance.
